# Supplementary material for: Epidemiology of aerophagia in children and adolescents: A systematic review and meta-analysis
Source: PLoS One. 2022 Jul 29;17(7):e0271494. doi: 10.1371/journal.pone.0271494 (PMC9337652; doi:10.1371/journal.pone.0271494)
Supplement: S1 Appendix — (DOCX) [file pone.0271494.s002.docx]

|  | **Supplementary Meterial 1: Search strategies** |
| --- | --- |
|  | 1. PubMed |
|  | Search Interface: NLM |
|  | PubMed-OLDMEDLINE (1910 – 2020) |
|  | The following filters were used |
| # | Search Strategy |
| #1 | "Aerophagy"[MeSh] OR "Aerophagy"[tiab] OR "Aerophagia"[MeSH] OR "Aerophagia"[tiab] OR "air swallowing"[MeSH] OR "air swallowing"[tiab] |
| #2 | ("Child"[Mesh] OR "Child, Preschool"[Mesh] OR "Infant"[Mesh] OR "Infant, Newborn"[Mesh] OR "Infant, Low Birth Weight"[Mesh] OR "Infant, Postmature"[Mesh] OR "Infant, Premature"[Mesh] OR "Adolescent"[Mesh] OR "Pediatrics"[Mesh] OR child*[tiab] OR infan*[tiab] OR newborn*[tiab] OR neonat*[tiab] OR baby[tiab] OR babies[tiab] OR pediatric*[tiab] OR paediatric*[tiab] OR adoles*[tiab] OR teen*[tiab] OR youth*[tiab] OR schoolchild*[tiab] OR preschool[tiab] OR pre-school[tiab] OR kid[tiab] OR kids[tiab] OR toddler*[tiab] OR juvenil*[tiab] OR teen*[tiab] OR pubescen*[tiab] OR puber*[tiab] OR prepubert*[tiab] OR school age*[tiab] OR schoolage*[tiab] OR elementary school[tiab] OR high school*[tiab] OR highschool*[tiab] OR kindergar*[tiab] OR boy[tiab] OR boys[tiab] OR girl*[tiab] OR minors[tiab] OR underag*[tiab] OR under ag*[tiab]) |
| #3 | ("Epidemiology"[Mesh] OR "Prevalence"[MeSH] OR "epidemiology" [Subheading] OR qpgs[tiab] OR rome III[tiab] OR rome iv[tiab] OR epidemiolog*[tiab] OR prevalen*[tiab]) |
| #3 | #1 AND #2 AND #3 |
|  |  |
|  |  |
|  |  |
|  |  |
|  | 1. **EMBASE** |
|  | Search Interface: Ovid |
|  | 1947 – March 2020 |
|  | The following filters were used |
| # | Search Strategy |
| #1 | ((Aerophagy or aerophagia) or (“air swallowing”)).mp |
| #2 | child/ or preschool child/ or infant/ or newborn/ or exp low birth weight/ or postmaturity/ or prematurity/ or adolescent/ or exp pediatrics/ or (child* or infant* or infancy or newborn* or neonat* or baby or babies or preschool or pre-school or pubescen* or teen* or adolescen* or puber* or prepubert* or juvenil* or p?ediatric* or youth* or schoolchild* or school age* or schoolage* or preschool or pre-school or elementary school or high school* or highschool* or kindergar* or boy or boys or girl* or minors or underag* or under ag* or kid or kids or toddler*).ti,ab,kw. |
| #3 | exp epidemiology/ or exp prevalence/ or epidemiology.fs. or (qpgs or rome III or rome iv or epidemiolog* or prevalen*).ti,ab,kw. |
| #4 | #1 and #2 and #3 |
|  |  |
|  |  |
|  | 1. **PsycINFO** |
|  | Search Interface: Ovid |
|  | The following filters were used |
| # | Search Strategy |
| #1 | pediatrics/ or (child* or infant* or infancy or newborn* or neonat* or baby or babies or preschool or preschool or pubescen* or teen* or adolescen* or puber* or prepubert* or juvenil* or p?ediatric* or youth* or schoolchild* or school age* or schoolage* or preschool or pre-school or elementary school or high school* or highschool* or kindergar* or boy or boys or girl* or minors or underag* or under ag* or kid or kids or toddler*).ti,ab,id. |
| #2 | Aerophagy/or aerophagia/or “Air swallowing”/ |
| #3 | "quality of life"/ or exp health/ or (quality of life or QoL or sickness impact profile* or value of life or life qualit* or HRQOL or HRQL or PedsQL or health scor* or health level or level of health).ti,ab,id. |
| #4 | #1 and #2 and #3 |
|  |  |
|  | 1. **Web of Science** |
|  | 1900 – March 2020 |
|  | Search Strategy |
| #1 | TS=(Aerophagy or aerophagia or “air swallowing”) |
| #2 | TS=(Child OR "Child, Preschool" OR Infant OR "Infant, Newborn" OR "Infant, Low Birth Weight" OR "Infant, Postmature" OR "Infant, Premature"OR "Adolescent" OR "Pediatrics" OR child* OR infan* OR newborn* OR neonat* OR baby OR babies OR pediatric*OR paediatric* OR adoles* OR teen* OR youth* OR schoolchild* OR preschool OR pre-school OR kid OR kids OR toddler* OR juvenil* OR teen* OR pubescen* OR puber* OR prepubert* OR school age* OR schoolage* OR elementary school OR high school* OR highschool* OR kindergar* OR boy* OR girl* OR minor* OR underag*OR under age*) |
| #3 | TS=(Epidemiology OR Prevalence OR “epidemiology.fs” OR qpgs OR rome III OR rome iv OR epidemiolog* OR prevalen*) |
| #4 | #1 AND #2 AND #3 |
|  |  |
