# Supplementary material for: Epidemiology of aerophagia in children and adolescents: A systematic review and meta-analysis
Source: PLoS One. 2022 Jul 29;17(7):e0271494. doi: 10.1371/journal.pone.0271494 (PMC9337652; doi:10.1371/journal.pone.0271494)
Supplement: Attachment — Submitted filename: Reviewer comments and answers.docx [file pone.0271494.s003.docx]

**Answers to Editorial and Reviewer Comments**

**Editorial Comments**

**Comment**

Please ensure that your manuscript meets PLOS ONE's style requirements, including those for file naming. The PLOS ONE style templates can be found at 
<https://journals.plos.org/plosone/s/file?id=wjVg/PLOSOne_formatting_sample_main_body.pdf> and 
<https://journals.plos.org/plosone/s/file?id=ba62/PLOSOne_formatting_sample_title_authors_affiliations.pdf>

**Response**

All are in accordance with PLOS ONE’s style requirements

**Comment**

We note that Figure 3 in your submission contain map images which may be copyrighted. All PLOS content is published under the Creative Commons Attribution License (CC BY 4.0), which means that the manuscript, images, and Supporting Information files will be freely available online, and any third party is permitted to access, download, copy, distribute, and use these materials in any way, even commercially, with proper attribution. For these reasons, we cannot publish previously copyrighted maps or satellite images created using proprietary data, such as Google software (Google Maps, Street View, and Earth). For more information, see our copyright guidelines: <http://journals.plos.org/plosone/s/licenses-and-copyright>.

**Response**

This figure was generated by the authors using the software ArcGIS 10.2 (Esri, Redlands, Canada). We used ESRI base map/base map outline. We have access to this software as it is a free access software. Therefore, our figures are original and do not need to obtain copyright permission from other authors, journals, or authorities.

**Comment**

In the methods, please describe how to provide the results of the publication bias analysis in the figures and state the specific test (Begg's or Egger's) used in the Methods section. Please also state the cut-off used to indicate heterogeneity using the I2 statistic in the Methods section.

**Response**

The necessary details were included in the method section as suggested by the editor.

**Comment**

In your Data Availability statement, you have not specified where the minimal data set underlying the results described in your manuscript can be found. PLOS defines a study's minimal data set as the underlying data used to reach the conclusions drawn in the manuscript and any additional data required to replicate the reported study findings in their entirety. All PLOS journals require that the minimal data set be made fully available. For more information about our data policy, please see <http://journals.plos.org/plosone/s/data-availability>.

**Response**

This is a systematic review and a meta-analysis. All the data included in this study are available in the public domain as published full text articles.

**Comment**

Please include captions for your Supporting Information files at the end of your manuscript, and update any in-text citations to match accordingly. Please see our Supporting Information guidelines for more information: <http://journals.plos.org/plosone/s/supporting-information>.

**Response**

Included at the end of the manuscript.

**Reviewer 1**

This manuscript is based on a systematic review and meta analysis of studies reporting aerophagia in children and adolescents. The systematic review appears to have been well conducted. I have a major concern regarding the manuscript and I have found several minor inaccuracies.

**Comment**

it is not appropriate to undertake a systematic review and meta analysis of the studies reporting the prevalence of aerophagia and to include data on risk factors and consequences. Studies on prevalence need samples representative of a defined population but representativeness is not an essential requirement to look at risk factors or consequences. It is likely that there is more literature reporting data on risk factors and consequences but these would not have met the inclusion criteria used in this review. In the manuscript there had been no synthesis of data related to the risk factors for aerophagia or its influence on the lives of children. Suggest confining the manuscript to the prevalence of aerophagia.

**Response**

Agreeing with the reviewer, we removed the data on risk factor and limited our manuscript to prevalence of aerophagia

**Comment**

I think "Global epidemiology" is rather misleading in the title for a review of the prevalence of a condition.

**Response**

We changed the title according to the reviewer comment.

**Comment**

Israel, Jordan, and Lebanon are listed under Europe in Table 1 but all three are in Asia.

**Response**

We thank the reviewer for pointing out this issue. It was corrected and Israel, Jordan, and Lebanon were included into Asian studies.

**Comment**

Reference number 27 is stated as the source of some of the data in Table 1, but this data has been extracted from reference number 16.

**Response**

We thank the reviewer for pointing out this error. The data were extracted from reference 16. The error was corrected.

**Comment**

Line 187 "There are five studies from four Asian countries[1-3, 13-15]' ...". This not correct.

**Response**

The studies were rearranged, and this error was corrected as pointed out by the reviewer.

**Comment**

There are six references (1, 2, 3, 13, 14, & 15) and in Table 1 under Asia six studies from four countries have been listed.

**Response**

This is correct. Three studies from Sri Lanka. The other countries were Iran, India, and Japan. Now we have added Jordan, Israel, and Lebanon to this list as suggested by the reviewer in his previous comment.

**Comment**

Line 196 in the manuscript mentions 14 studies but Table 2 has 19 studies.

**Response**

We thank the reviewer for pointing out this error. It was corrected to 19.

**Comment**

How was the data for figure 3 obtained? Is figure 3 really necessary?

**Response**

Each country had at least one prevalence rate for aerophagia. When there were more than one study providing prevalence, we calculated the average for the country through the meta-analysis. This was done for Sri Lanka and Colombia where we observed multiple prevalence rates. We believe that the prevalence map is necessary and if the reviewer is not disagreeing, we would like to keep it in the manuscript.

**Reviewer 2**

**Comment**

Although aerophagia in children is clinically less important comparing to other medical conditions, underlying pathophysiology can be important and needs further investigation. This study describes prevalence of aerophagia in children across the world, and discuss its effects on their life and possible underlying causes, such as stress and maltreatments. This study sends a message that aerophagia in children might have underlying causes and it should be investigated accordingly to prevent children from unnecessary stress and/or maltreatments.

**Response**

Thank you for your comment

**Comment**

I would suggest further research to assess super-gastric belching in children. Because supra-gastric belching in children has not been well documented yet, and it can also be related to stress factors.

**Response**

We have discussed the possibility of supra-gastric belching as an aetiological factor in the discussion stressing that it need to be looked at during evaluation.
